# Changes in fitness and fatness in Australian schoolchildren during the summer holidays: fitness lost, fatness regained? A cohort study

**DOI:** 10.1186/s12889-023-17009-4

**Published:** 2023-10-25

**Authors:** Tim Olds, Dorothea Dumuid, Emily Eglitis, Rebecca Golley, François Fraysse, Aaron Miatke, Grant R. Tomkinson, Amanda Watson, Mason Munzberg, Carol Maher

**Affiliations:** 1 Alliance for Research in Exercise, Nutrition and Activity (ARENA), Allied Health and Human Performance, University of South Australia, Adelaide, 5000 Australia; 2 Murdoch Children’s Research Institute, Parkville, 3052 Australia; 3 Caring Futures Institute, College of Nursing and Health Sciences, Flinders University, Bedford Park, 5042 Australia

**Keywords:** Shuttle run, Child, Summer, Holiday, Fitness, Fatness, Obesity, Use of time, Body Mass Index, Socioeconomic status

## Abstract

**Background:**

Emerging evidence suggests that children’s fatness increases and fitness declines at a greater rate during the summer holiday period, compared with the school year. The aim of this study was to compare rates of change in fitness and fatness over the in-term and summer holiday periods among Australian schoolchildren. A secondary aim was to explore whether rates of change differed according to the child’s sex, socio-economic status (SES), pubertal status and weight status.

**Methods:**

Children (n = 381) initially in Grade 4 (age 9) were recruited for this 2-year longitudinal study. Fatness (% body fat, BMI z-score, waist-to-height ratio) and fitness (20-m shuttle run and standing broad jump) were measured at the start and end of two consecutive years. Rates of change were calculated for the two in-school periods (Grades 4 and 5) and for the summer holiday period. Rates of change in fatness and fitness between in-school and holiday periods were compared, and differences in rates of change according to sex, socio-economic status, and weight status were explored.

**Results:**

During the holidays, percentage body fat increased at a greater rate (annualised rate of change [RoC]: +3.9 vs. Grade 4 and + 4.7 vs. Grade 5), and aerobic fitness declined at a greater rate (RoC − 4.7 vs. Grade 4 and − 4.4 vs. Grade 5), than during the in-school periods. There were no differences in rates of change for BMI z-score, waist-to-height ratio or standing broad jump. Body fatness increased faster in the holidays (relative to the in-school period) in children who are overweight and from low-SES families. Aerobic fitness declined more rapidly in the holidays in children who are overweight.

**Conclusion:**

This study highlights that during the summer holiday period, children experience greater increases in fatness and declines in fitness, with children who live with low-SES families and are overweight being more affected. The findings suggest the need for targeted interventions during this period to address these negative health trends.

**Trial registration:**

Australia New Zealand Clinical Trials Registry, identifier ACTRN12618002008202. Retrospectively registered on 14 December 2018.

**Supplementary Information:**

The online version contains supplementary material available at 10.1186/s12889-023-17009-4.

## Background

Physical fitness and body composition play a crucial role in the overall health and wellbeing of children [1, 2]. Poor physical fitness and increased fatness are associated with long-term negative health outcomes, including higher risks for cardiovascular diseases and Type 2 diabetes later in life [3, 4]. Understanding the factors that influence physical fitness and body composition, including temporal fluctuations such as the school summer holiday period, can help identify critical periods of risk and thereby inform targeted interventions to better address the growing challenge of childhood obesity.

Recent research suggests that increases in fatness and declines in aerobic fitness in children occur at a greater rate during the school summer holiday period compared to the school year [5–8]. One United States (US) study [9] also found that these negative health outcomes were more pronounced among children with overweight/obesity or from low socio-economic backgrounds. Therefore, the school summer holiday period has been identified as a high-risk period for unfavourable changes in body composition and physical fitness.

The “Structured Days Hypothesis” [10] offers an explanatory framework, suggesting that the relatively unstructured days of the summer holidays lend themselves more to obesogenic behaviours (less physical activity, more screen time, irregular sleep, excess intake of unhealthy food and drinks). This is in contrast to the structured days during the school period, which include physical education classes, activity opportunities at lunch and recess, healthy school meals/lunchboxes, limited screen time, and consistent bedtimes. Much of the current evidence on summer holiday weight gain and fitness losses originates from the US. Moreno and colleagues studied changes in children’s zBMI scores across the summer and school year over a five-year period and found an increase of 5.2 percentile points in the summer compared with a decrease by 1.5 percentile points over the school year [7]. Brusseau reported a school-based intervention which improved BMI and cardiovascular fitness during the school year; however, the children experienced significant increases in BMI and decreases in fitness over summer [11]. Interestingly, these negative changes in fitness and BMI were evident in both seven and twelve-week breaks, but intensified during longer school breaks [12]. Summer holiday durations differ significantly worldwide. For example, holidays are typically 5 weeks in Thailand [13] and Singapore [14], 9 weeks in France [15] and 11 weeks in Algeria [16]. The impact of shorter summer holidays on weight gain and fitness losses remains uncertain. In addition, the timing of major festive events may interact with summer holiday changes. As of now, no studies have examined summer holiday weight and fitness changes in southern hemisphere countries, where summer coincides with Christmas. Moreover, children’s summer activities vary significantly across different regions. In North America and Europe, children commonly attend summer camps [17] whereas these are relatively uncommon in many other world regions.

The aim of this study was to describe and compare rates of change in children’s fitness and fatness over the in-school and summer holiday periods in Australia, a southern hemisphere country with relatively short summer holidays (6 weeks) and relatively few summer camp offerings. A secondary aim was to explore whether rates of change differed according to the child’s sex, socio-economic status (SES), pubertal status and weight status.

## Materials and methods

### Participants

The participants in this study were drawn from the *Life on Holidays* study [18], a 2-year (wave 1: 2019–2020; wave 2: 2020–2021) longitudinal (cohort) study tracking changes in children’s fitness and fatness across the in-school and summer holiday periods. Children were recruited from 24 primary (elementary) schools in Adelaide, Australia. They were in Grade 4 (age 9 years at the time of enrolment). A stratified random sample was used to select schools. To achieve this, all primary schools in metropolitan Adelaide were divided into socio-economic tertiles (low, medium, and high) according to the Index of Community Socio-Educational Advantage [19], a socio-economic status indicator based on parental education, occupation, and school location. Schools were invited at random within tertiles, with a probability proportional to the number of students enrolled in each school. Once a school accepted the invitation, all children in Grade 4 were invited to take part. Recruitment continued until at least 100 children from each SES tertile had been recruited. Recruitment occurred in two waves, starting in 2019 and 2020. A total of 381 children were enrolled, with complete data available on 127–156 participants according to the outcome, partly due to COVID-related interruptions in data collection.

### Measures

The dependent variables were measures of fatness (percentage body fat, Body Mass Index (BMI) z-score and waist-to-height ratio) and fitness (maximal aerobic power, standing broad jump). Each child’s height was measured using a Seca 213 stadiometer (Seca, Hamburg, Germany), and weight and percentage body fat (*%BF*) using the InBody 270 Bioelectrical Impedance Analyser scales (InBody, Seoul, South Korea), without shoes and in light clothing. BMI was calculated from height and weight, with age- and sex-specific z-scores (*zBMI*) derived [20]. When compared to underwater weighing, the InBody provides a valid (r = 0.69–0.79 for children of this age) and reliable (CV_intra_ = 3%) estimate of body fat [21]. Waist girth was taken using a Lufkin W606 PM steel anthropometric tape (Michigan, USA) held at the midpoint between the iliac crest and the bottom of the bottom of the rib cage in the midline of the body. Waist girth was expressed as a percentage of height to normalise for body size (*Waist:Ht%*). Both waist circumference and BMI show high intra- and inter-rater reliability (> 0.88 and > 0.90 respectively [22]). V̇O_2max_ (ml/kg/min) was estimated using the 20-m shuttle run test (20mSRT), a widely used aerobic fitness assessment where individuals run back and forth between two lines 20 m apart, following audio cues that gradually increase in speed, until they can no longer maintain the pace [23]. The performance scores from the 20mSRT were then converted to; V̇O_2max_ values using the equation of Nevill et al. [24]. The 20mSRT has high to very high test-retest reliability (r = 0.78–0.93) [25] and good criterion validity (r = 0.78) compared to gas-analysed graded exercise tests [26]. Explosive strength was assessed using the standing broad jump (*SBJ* in cm [27]). This measure was included alongside the 20mSRT to provide a more comprehensive understanding of children’s health-related fitness levels. The SBJ is an excellent general measure of explosive strength [28] that has excellent health-related predictive validity and is recommended for school-based testing [29].The child jumped as far forward as possible from a standing position, swinging their arms and bending their knees before take-off. The best of three jumps was retained for analysis. In children of this age, the standing broad jump test has very high test-retest reliability (ICC = 0.88) [30]. This test is also closely related to other lower body muscular strength tests (R^2^ = 0.83–86), as well as upper body muscular strength tests (R^2^ = 0.69–0.85) [28].

The *covariates* were sex, SES, pubertal status, and weight status. Sex and SES were obtained via a one-off parent questionnaire at baseline. SES was quantified based on parent reported occupation, household income and highest education level (for both parents). From these, a composite SES z-score was derived, based on the procedure outlined in Gibbings, Blakemore and Strazdins [31].

Pubertal status was measured using The Pubertal Development Scale [32]. For this, parents were asked to report on their child’s stage of pubertal development based on a number of physical indicators including the development of body hair, occurrence of growth spurt, and changes in complexion. All questions were answered on a 4-point scale (1 = has not begun, 2 = has barely started, 3 = is definitely underway, 4 = growth or development is definitely complete) [32]. Children’s weight status was obtained by categorising their BMI as either underweight, healthy weight, overweight, or obese using the International Obesity Taskforce criteria [33]. As only 5 participants (1%) were classified as underweight, these were included in the normal weight category.

### Bias

Numerous efforts were made to minimise study biases. In particular, a randomised stratified sampling methodology was used. Outcomes were gathered using high-quality tools and protocols with established reliability and validity. Research personnel were thoroughly trained, and participant retention was maximised through the use of multiple reminders and follow ups and a yearly incentive for participants to remain engaged in the study.

### Sample size justification

Full details of the power calculation are provided in the study protocol [18]. Briefly, a target sample size of n = 225 completers would provide 80% power to detect the hypothesised difference of change in % body fat of 0.6% per year between the in-school and holiday period. The sample size was inflated to account for study drop out.

### Analysis

**Fig. 1:**
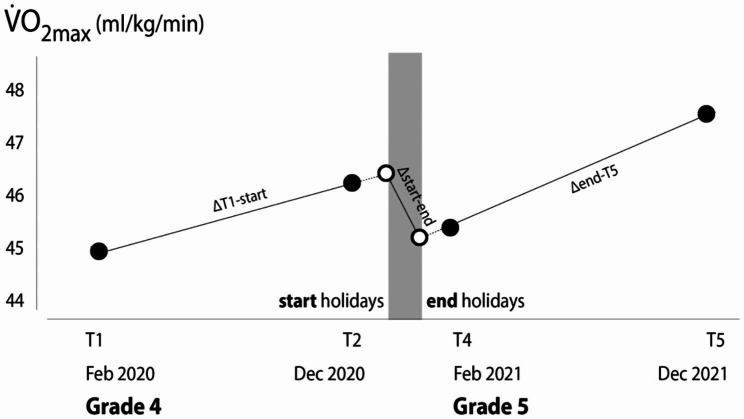
Schematic of data treatment to extrapolate the rates of change in fatness and fitness across Grade 4, Grade 5 and the holiday period. Outcomes (here, estimated VO_2max_) were measured at the start (T1) and end (T2) of Grade 4, and at the start (T4) and end (T5) of Grade 5. Values were then forward- (Grade 4) or back-extrapolated (Grade 5) to estimate the values at the start and end of the holidays. Rates of change between timepoints, indicated by the slopes of the segments, were derived by dividing the calculated change by the number of intervening days, and then annualised. ∆T1-start = rate of change from T1 to start of holidays; ∆Start-End = rate of change from start to end of holidays; ∆End-T5 = rate of change from end of holidays to T5

Figure 1 shows the process of data treatment. Participants were measured at four timepoints: first term in Year 4 (T1; February to April 2019 for Wave 1/2020 for Wave 2), last term in Year 4 (T2; October to December 2019 for Wave 1/2020 for Wave 2), first term in Year 5 (T4; February to April 2021 for Wave 1/2022 for Wave 2), and last term in Year 4 (T5; October to December 2021 for Wave 1/2022 for Wave 2). There was a measurement point during the summer holidays (T3), but this was irrelevant to this study. For each of the five outcomes, the rate of change from T1 to T2 was extrapolated to the start of the summer holidays (typically within a month of the November-December measurements) to estimate what the value would have been at the start of the holidays. Similarly, the change from T4 to T5 was back-extrapolated to the end of the summer holidays (typically within a month of the February-April measurements) to estimate what the value would have been at the end of the holidays. Rates of change were then calculated for each of the outcome variables for the periods T1 to start of holidays (∆T1-start), start to end of holidays (∆start-end), and end of holidays to T5 (∆end-T5), by dividing the change by the number of days for each period. These were then expressed as rate of change per year for ease of understanding.

Multi-level models were carried out in R [34] using the *nlme* package [35] to test whether the rate of change in outcomes was different during holidays compared with the in-school periods (the R script is provided in Supplementary file 1). The dependent variable was the daily rate of change in outcome (%body fat, *z*BMI, Waist:Ht%, V̇O_2max_ and standing broad jump). The independent variable (fixed effect) was the timespan (Grade 4, Grade 5 or holidays), with contrasts set to compare the holiday period to each of the in-school periods (i.e., a positive beta for the holiday-Grade 4 contrast would indicate a higher rate of change in the outcome variable during the holidays than during Grade 4). Random effects (intercepts) were included to account for the repeated measures within participants, within schools, within study waves. Model diagnostic plots showed that the assumption of homogeneity of variance of residuals was violated – variance was greater during holidays than during the in-school periods. To accommodate the heteroscedasticity, we allowed for independent spread in the variance of the timespan variable using the *varIdent* variance structure in *nlme*. To test for the moderation effects of sociodemographic variable (sex, SES, pubertal status and weight status), interaction effects between these variables and the timespan variable were included in a second set of models. All betas were expressed as annualised rates of change (daily rate of change * 365), and plotted to aid interpretation. An alpha of 0.05 was used to denote statistical significance.

## Results

Participant characteristics are shown in Table 1. A total of 381 children enrolled for the study, but 23 dropped out before the study began. Complete data on outcomes at all four time-points were available for n = 133 for %BF, n = 127 for zBMI, n = 129 for Waist:Ht%, n = 132 for V̇O_2max_, and n = 156 for SBJ.

**Table 1:** Participant characteristics

Participants (n)	156	
Age at baseline (years, mean (SD))	9.4	(0.3)
**Sex (n = male (%))**	65	(42)
**SES (mean (SD))**	0.01	(0.63)
**Puberty (%)**		
Prepubertal	47	(34.8)
Early pubertal	35	(25.9)
Mid-pubertal	53	(39.8)
**BMI category (%)**		
Underweight	3	(2)
Normal weight	94	(62)
Overweight	36	(24)
Obese	18	(12)

Data are shown for the outcome with the largest sample size (n = 156 for SBJ)

BMI = Body Mass Index; SES = socio-economic status

**Fig. 2:**
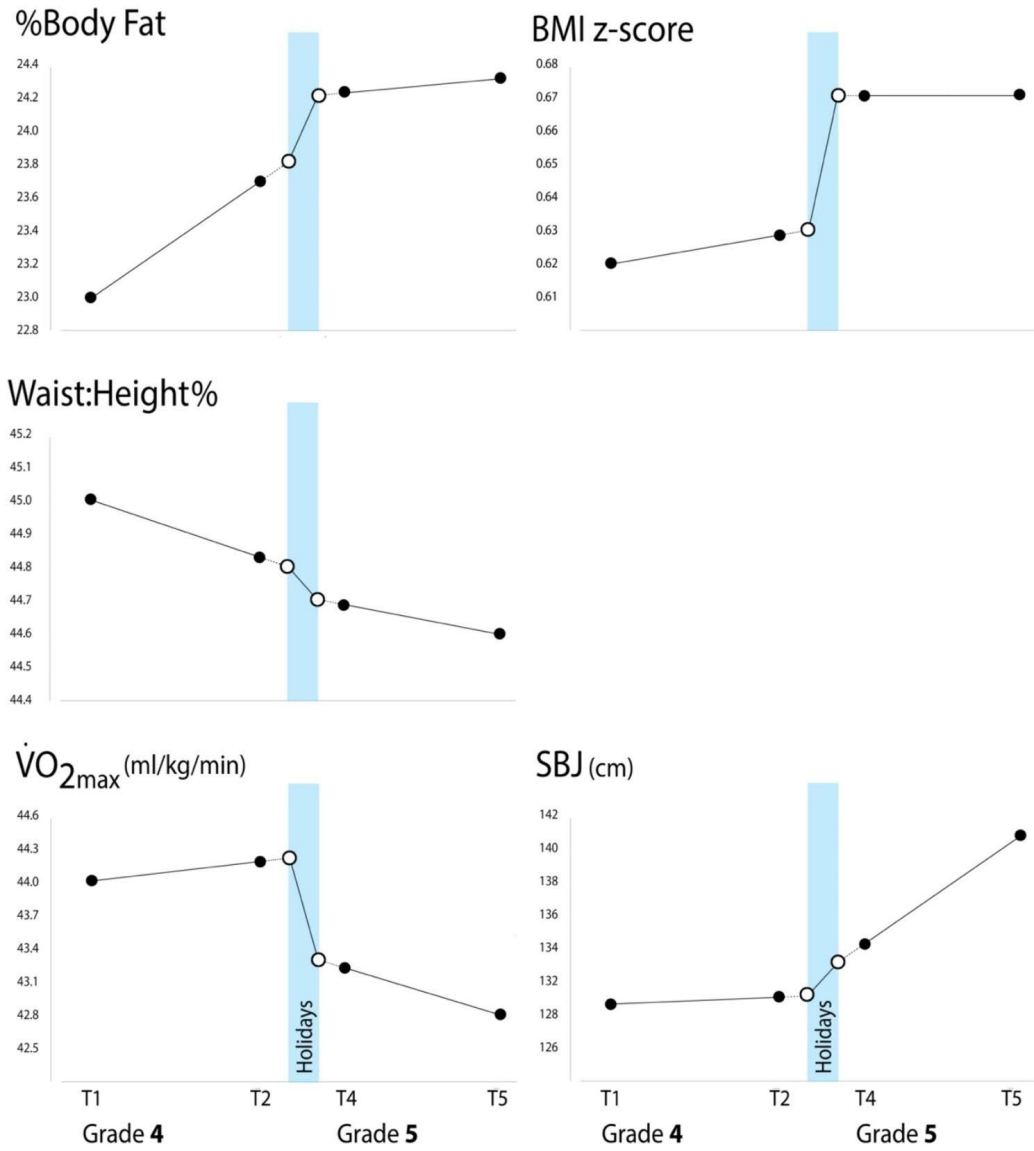
Visualisation of the observed rates of change in fatness and fitness across Grade 4, Grade 5 and the holiday period. Filled symbols represent measured values and empty symbols extrapolated values

Table 2; Fig. 2 show the values of the dependent variables at each timepoint, and the annualised rates of change. Children generally got fatter over the 2-year period (%BF increased by 6%, and zBMI by 8%), although Waist:Ht decreased marginally. Aerobic fitness declined by 3% while explosive strength improved by 9%.

**Table 2:** Observed rates of change (means, SDs) for fatness and fitness outcomes across the study timepoints.

	T1	Start of Holidays	End of Holidays	T5	∆T1-Start	∆Start-End	∆End-T5
**%BF**	23.0	23.8	24.2	24.3	+ 1.0	+ 3.6	+ 0.2
n = 133	(8.3)	(8.1)	(8.9)	(8.4)	(3.8)	(21.6)	(4.1)
**zBMI**	0.62	0.63	0.67	0.67	+ 0.02	+ 0.32	+ 0.00
n = 127	(1.16)	(1.15)	(1.22)	(1.14)	(0.45)	(2.69)	(0.42)
**Waist:Ht%**	45.0	44.8	44.77	44.6	–0.4	–0.7	–0.2
n = 129	(5.8)	(5.9)	(6.5)	(5.9)	(4.0)	(2.6)	(4.3)
**VO**_**2max**_	44	44.2	43.3	42.8	+ 0.1	–3.7	–0.7
n = 132	(3.2)	(4.2)	(4.0)	(3.9)	(4.2)	(17.2)	(4.4)
**SBJ**	128.5	130.5	133.1	140.5	+ 2.2	+ 9.1	+ 5.8
n = 156	(19.9)	(22.3)	(23.3)	(22.1)	(19.0)	(124.1)	(18.6)

%BF = percentage body fat; ∆T1-start = annualised rate of changed from T1 to start of holidays; ∆Start-End = annualised rate of change from start to end of holidays; ∆End-T5 = annualised rate of change from end of holidays to T5; SBJ = standing broad jump (cm); T1 = timepoint 1 (February-April); T5 = timepoint 5 (November-December); V̇O_2max_ = maximal aerobic power (ml/kg/min); Waist:Ht% = waist-to-height ratio (as a percentage); zBMI = BMI z-score

Percentage body fat increased at a faster rate during the holidays than during the in-school periods. The rate of increase in %BF was significantly higher in the holidays than in the Grade 5 school year (by + 4.7%BF/year; p = 0.030; Table 3), though the difference, which was of a similar magnitude, was not significant for the Grade 4 school year (+ 3.9%BF/year; p = 0.076). Figure 2 suggests that aerobic fitness improved slightly in Grade 4, decline rapidly during the holidays, and declined slowly in Grade 5. Statistical analyses (Table 3) contrasting the rate of change during the holidays versus the other timepoints showed that aerobic fitness declined more rapidly during the holidays than during the in-school periods. The annualised rate of decline in V̇O_2max_ was significantly greater in the holidays compared to both the Grade 4 (by 4.7 ml/kg/min/year; p = 0.010) and Grade 5 school years (by 4.4 ml/kg/min/year; p = 0.015). There were no significant differences in rates of change across periods for zBMI, Waist:Ht%, or SBJ.

**Table 3:** Annualised rates of change in fatness and fitness metrics during the holiday periods compared to in-school periods

Predictor	Versus	%BF	p	zBMI	p	Waist:Ht%	p	VO_2max_	p	SBJ	p
**Time**	**Holidays vs Grade 4 in-school**	3.9	0.08	0.2	0.54	-0.3	0.9	-4.7	**0.01**	17.2	0.12
		(-0.4 to 8.1)		(-0.3 to 0.7)		(-4.4 to 3.9)		(-8.3 to -1.2)		(-4.2 to 38.6)	
	**Holidays vs Grade 5 in-school**	4.7	**0.03**	0.2	0.52	-0.4	0.86	-4.4	**0.015**	10.6	0.33
		(0.5 to 9.0)		(0.3 to 0.7)		(-4.6 to 3.8)		(-8.0 to -0.9)		(-10.8 to 32.0)	

The table depicts the differences in annualised rates of change (and 95% CIs) during the holiday period compared to the two in-school periods (School Year Grade 4 and School Year Grade 5). For example, during the holiday period, body fat percentage increased at a rate of 4.7%BF/year more than during Grade 5.

BF = percentage body fat; SBJ = standing broad jump (cm); V̇O_2max_ = maximal aerobic power (ml/kg/min); Waist:Ht% = waist-to-height ratio (as a percentage); zBMI = BMI z-score. Note: p < 0.05 shown in boldface

We next explored whether differences in rates of change (holidays vs. school years) varied according to socio-demographic characteristics (sex, SES, pubertal status, weight status; Table 4).

**Table 4:** Comparative annuaslised rates of change in fatness and fitness metrics by socio-demographic categories: holiday vs. in-school periods

Predictor		%BF	P	zBMI	p	Waist:Ht%	p	VO_2max_	p	SBJ	p	
Time*Sex	Holidays vs Grade 4*Male	-2.0	0.75	0.6	0.43	-8.2	0.20	7.4	0.18	-14.2	0.69	
***Ref*** **= Female**		(-14.1 to 10.2)		(-0.9 to 2.2)		(-20.6 to 4.2)		(-3.4 to 18.1)		(-84.6 to 56.2)		
	Holidays vs Grade 5*Male	-3.7	0.55	0.3	0.69	-7.0	0.27	8.1	0.15	-14.6	0.68	
		(-15.9 to 8.4)		(-1.2 to 1.9)		(-19.5 to 5.5)		(-2.8 18.9)		(-85.0 to 55.8)		
Time*SES	Holidays vs Grade 4*SES	-7.5	**0.045**	-0.7	0.13	-1.5	0.69	-0.6	0.84	-5.0	0.79	
		(-14.8 to -0.2)		(-1.6 to 0.2)		(-8.7 to 5.8)		(-6.5 to 5.3)		(-41.4 to 31.4)		
	Holidays vs Grade 5*SES	-9.4	**0.01**	-0.9	**0.04**	-2.9	0.44	-2.6	0.38	-6.1	0.74	
		(-16.8 to -2.1)		(-1.8 to -0.0)		(-10.2 to 4.4)		(-8.6 to 3.3)		(-42.5 to 30.3)		
Time*	Holidays vs Grade 4* Early	-12.8	**0.02**	-1.4	**0.046**	-12	**0.04**	-5.4	0.25	-9.2	0.75	
**Puberty**		(-23.9 to -1.8)		(-2.7 to -0.0)		(-23.2 to -0.8)		(-14.6 to 3.9)		(-65.0 to 46.5)		
***Ref*** **= Mid**												
	Holidays vs Grade 5* Early	-10.8	0.06	-1.2	0.08	-10.9	0.06	-4.6	0.33	3.3	0.91	
		(-21.9 to 0.3)		(-2.5 to 0.1)		(-22.2 to 0.5)		(-13.9 to 4.7)		(-52.4 to 59.0)		
	Holidays vs Grade 4* Pre	0.2	0.98	-0.5	0.58	12.2	0.08	-12.9	**0.03**	50	0.19	
		(-13.0 to -13.3)		(-2.1 to 1.2)		(-1.5 to 25.9)		(-24.5 to -1.3)		(-25.5 to 125.4)		
	Holidays vs Grade 5* Pre	3.2	0.64	0.0	0.98	11.6	0.10	-13.2	**0.03**	61.5	0.11	
		(-10.1 to 16.4)		(-1.7 to 1.6)		(-2.2 to 25.4)		(-24.8 to -1.5)		(-13.9 to 137.0)		
Time*	Holidays vs Grade 4*	11.2	**0.03**	0.9	0.14	3.1	0.55	-11.3	**0.01**	3.48	0.90	
**Weight Status**	**Overweight**	(1.3 to 21.1)		(-0.3 to 2.1)		(-7.1 to 13.3)		(-20.2 to 2.4)		(-50.2 to 57.1)		
***Ref*** **= Normal**	Holidays vs Grade 5* Overweight	11.9	**0.02**	0.9	0.13	3.1	0.55	-11.8	**0.01**	5.9	0.830	
		(0.19 to 21.8)		(-0.3 to 2.1)		(-7.1 to 13.3)		(-20.8 to -2.8)		(-47.8 to 59.5)		
	Holidays vs Grade 4* Obese	4.4	0.53	0.2	0.82	4.9	0.47	-4.0	0.56	8.8	0.81	
		(-9.2 to 17.9)		(-1.4 to 1.8)		(-8.6 to 18.5)		(-17.1 to 9.2)		(-62.2 to 79.7)		
	Holidays vs Grade 5* Obese	6.1	0.38	0.4	0.61	7.1	0.31	-7.8	0.25	8.0	0.83	
		(-7.5 to 19.7)		(-1.2 to 2.0)		(-6.5 to 20.7)		(-21.0 to 5.4)		(-63.0 to 78.9)		

The table delineates the variations in annualised rates of change (accompanied by 95% CIs) during the holiday period, contrasted against the Grade 4 and Grade 5 in-school periods, segmented by socio-demographic categories. For instance, in the context of body fat percentage (%BF), the rate of change during the holidays compared to the Grade 5 in-school period was notably higher (p = 0.020) in overweight children by + 11.9%BF/year when compared to normal-weight children (who serve as the reference category). In terms of Socioeconomic Status (SES), a 1 SD increase in the SES z-score was associated with a decrease of 9.4%BF/year in the rate of change in %BF during holidays versus the Grade 5 in-school period.

Models were mutually adjusted for all covariates, and random intercepts were used to account for nesting of observations within participants, within schools, within study waves. Note: p < 0.05 shown in boldface

Abbreviations: SES = socioeconomic status; Wt status = weight status; %BF = percentage body fat; SBJ = standing broad jump (cm); V̇O_2max_ = maximal aerobic power (ml/kg/min); Waist:Ht% = waist-to-height ratio (as a percentage); zBMI = BMI z-score

### Sex

Sex did not moderate differences in rates of change for any outcome.

### Socio-economic status

SES moderated differences in the rate of change in %BF and zBMI. Overall, children from lower SES families showed greater increases in fatness over the holidays compared to children from higher SES families. Every 1 SD increase in SES was associated with a 7.5%BF/year greater difference in the rate of change during the in-school period in Grade 4 compared to the holidays (p = 0.045), and a 9.4%BF/year greater difference for Grade 5. Similarly, a 1 SD increase in SES was associated with a 0.9 SD/year greater relative difference in rates of change in zBMI in Grade 5 vs. the holidays (p = 0.040), although the relative difference of + 0.7 SD/year was not significant for Grade 4. Socio-economic status did not moderate differences in the rates of change of Waist:Ht%, SBJ, or V̇O_2max_.

### Pubertal status

Pubertal status moderated differences in rates of change in all fatness measures. Children who were early pubertal in Grade 4 showed relatively lower increases in all fatness measures over the holidays compared to children who were mid-pubertal (p = 0.023–0.046). In Grade 5, these differences were similar but did not reach statistical significance. Children who were pre-pubertal, however, showed relatively faster declines in aerobic fitness during the holiday periods compared to children who were mid-pubertal (p = 0.027–0.029). Pubertal status did not moderate differences in rates of change in SBJ performance.

### Weight status

Weight status moderated differences in rates of change in %BF. Compared to children of normal-weight, children who were overweight (but not obese) increased %BF relatively faster in the holiday periods than in both in-school periods (p = 0.020–0.027). The corresponding rate of decline in aerobic fitness for children who were overweight (but not obese) was higher in the holiday period (p = 0.010–0.013). Weight status did not moderate differences in the rates of change of zBMI, Waist:Ht% or SBJ.

## Discussion

### Main findings

The focus of this study was to compare rates of change in children’s fitness and fatness between in-school and summer holiday periods, and to explore whether differences in rates of change in these outcomes were moderated by sex, SES, pubertal status, or weight status. During the holiday period, there was a significant increase in the rate of change of percentage body fat and a significant increase in the rate of decline of aerobic fitness, indicating differential increases in fatness and decreases in fitness in the holiday period. However, there were no differences in rates of change for BMI z-score, waist-to-height ratio, or standing broad jump. Some differences in rates of change in fitness and fatness were moderated by socio-demographic characteristics. In general, children who were overweight and from lower-SES families exhibited relatively faster increases in fatness and declines in fitness in the holidays compared to the in-school period.

The study’s finding of summer holiday weight gain is generally consistent with previous studies [7, 36, 37]. Similarly, studies from Sallis et al. [38] and Fu et al. [39] have reported summer holiday fitness losses. Interestingly, the size of the effects in this study were generally smaller than those reported in European and North American studies. Furthermore, the effects in our study were not consistent across all measures of fatness and fitness, which also contrasts with previous literature. It is possible that Australia’s relatively short summer holidays (which are only half to one-third as long as those in North America and Europe) may underpin these differences in findings.

Few previous studies have examined whether summer holiday weight gain and fitness losses differ according to sociodemographic and anthropometric characteristics [9, 40]. Consistent with Franckle et al. [9] we found that weight gain and fitness losses appeared to be greater for children from low-SES families. Other studies have identified SES gradients in other summer holiday deficits, such as academic losses, and that the differential widens over time [41]. It is possible that economic and environmental barriers faced by low-SES households (inability to purchase nutritious foods, less access to safe neighbourhoods and organised extracurricular activities to promote adequate physical activity [40]) are exacerbated during the school holidays when children spend more time at home. While our study only measured changes over one summer holiday period, it is conceivable that the SES differential identified in this study may accumulate over time, contributing to the recognised higher rates of children who were overweight or obese from low SES families [34].

Similar to Moreno et al. [7], our results suggested that summer holiday weight gain and fitness loss was worse for children who were overweight, relative to those who were normal weight. Interestingly, this pattern was not confirmed for children who were obese. The model beta values suggested that the magnitude of weight gain and fitness loss was larger for children who were obese relative to children of normal weight, however the results were not statistically significant. It is possible that the relatively small number of children with obesity (n = 18) meant that this comparison was underpowered.

Interestingly, patterns for summertime changes in fatness and fitness were not consistent for the various markers. Generally speaking, results were significant when fatness was considered based on %BF (and mostly consistent based on zBMI), but not when considered based on waist:height. It is likely that %BF measured by bioimpedance is a more sensitive measure of adiposity than zBMI and waist:height. Furthermore, it is possible that changes in body shape associated with puberty may have contributed to the lack of significant findings for the waist:height variable.

Similarly, clear fitness losses were apparent in V̇O_2max_ estimated from the SRT, whilst there were summertime improvements in standing broad jump performance. For the standing broad jump, the rate of improvement across summer was similar to that observed during the subsequent Grade 5 school year. Previous research has reported that standing broad jump performance improves each year by 8.3–9.1 cm [42], which is very similar to the 7.2 cm p.a. increase observed in this study. The reasons underpinning the differential in patterns of change in aerobic fitness versus muscular fitness are unclear. One explanation is that muscular fitness may be relatively more stable than aerobic fitness. However, previous research has identified Australian children do less moderate-to-vigorous physical activity in the holidays compared to school time [43], which may explain the loss of aerobic fitness over the summer. In contrast, it is possible that children continue to engage in sufficient muscle-strengthening activities over the summer to maintain their muscle fitness, but insufficient aerobic activities to maintain their aerobic fitness. Furthermore, age-related growth, pubertal development and motor skill development may contribute to improvements in muscular fitness despite the relatively smaller dose of MVPA during the holidays.

It is interesting to consider that the significant increases in percentage body fat and decreases in aerobic fitness may even be directly related. Given that the 20mSRT is performed under gravity where children carry their entire body mass, a holiday-related increase in BMI should reduce 20mSRT performance. Fat mass per se does not affect aerobic fitness but increased fat mass could reduce 20mSRT performance and our estimate of V̇O_2max_, which uses body mass as an input. Because fat mass is partly metabolically inactive and constitutes an additional load to carry, increased fat mass could reduce V̇O_2max_ (mL/kg) by inflating the denominator [44]. Therefore, increased fatness could reduce V̇O_2max_ when expressed as mL/kg even if true aerobic fitness (i.e., absolute V̇O_2max_ in the numerator) increased. Any holiday-related decline in physical activity levels that increased fat mass could in turn reduce V̇O_2max_ in mL/kg (and 20mSRT performance).

### Strengths and limitations

Key strengths of the current study are its longitudinal design spanning two school years. Furthermore, it used the highest quality measures of fatness and fitness possible for collection in a school setting. Relatively few studies of children’s summer holiday fatness and fitness have been conducted outside the US, and none in the southern hemisphere or in countries with relatively short summer holidays, meaning this study makes a highly valuable contribution to the international literature.

Limitations must also be acknowledged. The COVID-19 pandemic commenced approximately halfway through data collection. This led to some data loss (because some schools wouldn’t permit data collection visits to go ahead) and also likely contributed to participant dropout, which was considerable. This is likely to have negatively affected the study’s power, and may also impact the generalisability of findings. Finally, we chose to study children across Year 4 and 5 on the basis that they were older enough to reliably complete fitness tests, and so that the likelihood of them changing schools would be minimised (children attending government schools in South Australia transition to high school at the end of Year 6, though a considerable portion choose to transition to private schools for high school, with many transitioning at the end of Year 5). However, we acknowledge that children’s bodies change rapidly at this age, which may make it harder to detect summer-holiday-related changes in fatness and fitness. Changes across the 2-year period are overlaid on expected age-related changes in growth and development. At this age, %BF increases on average by 0.3–0.8% p.a [45], somewhat less than the 0.8% p.a. increase in this study. Cross-sectional data indicate that V̇O_2max_ decreases by 1.2–1.6 ml/kg/min/year [46], somewhat more than the 0.7 ml/kg/min/year in the current study.

### Implications

If the holiday environment leads to increases in fatness and decreases in aerobic fitness, there are potential policy implications. Interventions targeted at the holiday period (such as summer camps and programs which offer a mix of physical and learning activities), at the home environment, or at effectively extending the in-school environment (such as shortening the holiday period) may provide the structured day needed to prevent weight gain and losses in aerobic fitness. Further, many sporting competitions stop during the summer holidays (e.g., netball, basketball) while others (e.g., little athletics) have a short Christmas-New Year break. Perhaps an emphasis on moderate-to-vigorous aerobic activities like swimming, bike riding, running, and dancing, as recommended in national physical activity guidelines, may help minimise holiday-related declines in aerobic fitness, particularly while competitive sports are in a hiatus.

There is accumulating evidence that interventions of this sort of structured programming have been effective in North America and Europe, where summer camps are common [47]. US studies have found summer camps to increase moderate- to vigorous- physical activity levels and steps, and reduce sedentary time [48] to reduce body fat and increase aerobic fitness [49], and to significantly reduce the risk of obesity in the subsequent year [50]. A nutrition- and fitness-focused 6-week summer day camp reduced weight and waist-to-height ratio among children who were overweight or obese [51]. Recently there has been some discussion around establishing a culture of summer camps in Australia, where they are much less common [52].

Family-based interventions are difficult during the holiday diaspora, but a recent systematic review [53] showed small-to-moderate benefits. Another systematic review found family-based interventions to be more effective than school-based interventions for reducing obesity in children who were of primary-school-age [54].

## Conclusion

In summary, this study provides important insights into the differential rates of change in children’s fitness and fatness during in-school and summer holiday periods. Our findings suggest that during the holiday period, there is a significant increase in the rate of change of percentage body fat and a significant decline in fitness. Furthermore, we found that socio-demographic characteristics such as SES and weight status moderated the rates of change in fitness and fatness, with children who were overweight and from lower-SES families exhibiting relatively faster increases in fatness and declines in fitness during the holiday period compared to the in-school period. These findings have important implications for policymakers and public health practitioners, highlighting the need for targeted interventions to address the summer holiday deficits in children’s fitness and fatness, particularly among low-SES and overweight populations. Overall, this study contributes to the international literature on children’s summer holiday fatness and fitness, and underscores the importance of longitudinal studies using high-quality measures of fitness and fatness in school settings.

### Electronic supplementary material

Below is the link to the electronic supplementary material.

Supplementary Material 1

## Data Availability

Data analysed during this study will be made available upon reasonable request to the corresponding author Carol Maher by emailing her at carol.maher@unisa.edu.au.
