# Supplementary material for: Changes in fitness and fatness in Australian schoolchildren during the summer holidays: fitness lost, fatness regained? A cohort study
Source: BMC Public Health. 2023 Oct 25;23:2094. doi: 10.1186/s12889-023-17009-4 (PMC10601165; doi:10.1186/s12889-023-17009-4)

R Codes for Changes in fitness and fatness in Australian schoolchildren during the summer holidays Fitness lost, fatness regained? A cohort study’

Olds et al

Updated 05/09/2023

```
#load packages (they need to be installed first)
require(readxl)
require(dplyr)
require(ggplot2)
require(tableone)
require(MASS)
require(tidyr)
require(nlme)
require(lme4)
require(lmerTest)
require(sjPlot)
require(lattice)
require(cowplot)
require(emmeans)

#import dataset
d = read_xlsx("Dataset.xlsx")

#select required variables
d=d %>% dplyr::select(id, wave, schoolid, sex, "T1 Age", sep, Puberty, "T1 zBMI",
   "dd BF T2-T1"  , "dd BF T5-T4" , "dd BF Hols",
   "dd zBMI T2-T1","dd zBMI T5-T4" , "dd zBMI Hols",
   "dd WHt T2-T1", "dd WHt T5-T4" , "dd WHt Hols",
   "dd SBJ T2-T1", "dd SBJ T5-T4" , "dd SBJ Hols",
   "dd VO2maxT2-T1", "dd VO2max T5-T4" ,"dd VO2max Hols")

#look at the first few rows of data
#the variables starting with "dd" are operationalised as daily rates of change
head(as.data.frame(d))
```

	id <chr>	wave <dbl>	schoolid <dbl>	sex <chr>	T1 Age <dbl>	sep <dbl>	Puberty <chr>
1	LOH001C	1	1	M	9.11	NA	NA
2	LOH002C	1	1	F	9.34	0.70	Prepubertal
3	LOH003C	1	1	M	9.44	NA	NA

4	LOH004C	1	1	M	9.28	0.33	Prepubertal
5	LOH005C	1	1	F	9.66	0.05	Early Pubertal
6	LOH006C	1	1	F	9.35	-0.35	Prepubertal

6 rows | 1-8 of 24 columns

```
#make BMI categories at time 1
d$bmi.cat.t1=cut(d$`T1 zBMI`,
                breaks=c(-5,-2, 1, 2, 5),
                labels=c("UW","NW", "OW","OB"))

#alternate categories that puts UW and NW together
d$bmicat.t1=cut(d$`T1 zBMI`,
                breaks=c(-5, 1, 2, 5),
                labels=c("NW (incl UW)", "OW","OB"))

#largest analytical sample for characteristics table (those who have standing broad jump)
tabl = d%>%dplyr::select(id, school1="dd SBJ T2-T1", school2="dd SBJ T5-T4", holidays="dd SBJ Hols")
tabl=(na.omit(tabl))#only keep those that don't have missing data
chartab=d %>% dplyr::filter(id%in%tabl$id)
#select the sample descriptives, which will be summarised in Table 1
ch= chartab %>% dplyr::select(id,"T1 Age", sex, sep, Puberty, bmi.cat.t1)
```

Descriptives table of largest sample (n=156 for SBJ in Table 1)

```
CreateTableOne(vars=c(names(ch[-1])), data=ch)
```

```
##
##                        Overall
##   n                     156
##   T1 Age (mean (SD)) 9.38 (0.29)
##   sex = M (%)          65 (41.7)
##   sep (mean (SD))    0.01 (0.63)
##   Puberty (%)
##      Early Pubertal    35 (25.9)
##      Midpubertal       53 (39.3)
##      Prepubertal       47 (34.8)
##   bmi.cat.t1 (%)
##      UW                 3 ( 2.0)
##      NW                94 (62.3)
##      OW                36 (23.8)
##      OB                18 (11.9)
```

```
#set up contrasts for "when" - want all comparisons to be against holidays.
#ie. model estimates will be for holidays vs in term periods.
mat <- rbind(c(1, -1, 0),
             c(1, 0, -1))
(cMat <- ginv(mat))
```

```
##              [,1]       [,2]
## [1,]  0.3333333  0.3333333
## [2,] -0.6666667  0.3333333
## [3,]  0.3333333 -0.6666667
```

```
######Set up data for %body fat analyses
#select required variables
bfdata = d %>% dplyr::select(id, wave, schoolid, sex, sep, Puberty, bmicat.t1,
                      school1="dd BF T2-T1"  , school2="dd BF T5-T4" , holidays="dd BF Hols")
#set up factor variables
bfdata$wave=as.factor(bfdata$wave)
bfdata$schoolid=as.factor(bfdata$schoolid)
bfdata$sex=as.factor(bfdata$sex)
bfdata$Puberty=as.factor(bfdata$Puberty)
bfdata$id = as.factor(bfdata$id)
bfdata$bmicat.t1=as.factor(bfdata$bmicat.t1)
#set reference levels
bfdata$sex=relevel(bfdata$sex, ref="F")
bfdata$Puberty=relevel(bfdata$Puberty, ref="Midpubertal")
bfdata$bmicat.t1=relevel(bfdata$bmicat.t1, ref="NW (incl UW)")

#make long dataset where repeated observations within individuals go down the rows
longbf = gather(bfdata, when, BF_ROC, school1:holidays)
longbf$when=as.factor(longbf$when)
#have a look at long dataset
head(longbf%>%arrange(id),12)
```

id <fct>	wave <fct>	schoolid <fct>	sex <fct>	sep <dbl>	Puberty <fct>	bmicat.t1 <fct>
LOH001C	1	1	M	NA	NA	NA
LOH001C	1	1	M	NA	NA	NA
LOH001C	1	1	M	NA	NA	NA
LOH002C	1	1	F	0.70	Prepubertal	OW
LOH002C	1	1	F	0.70	Prepubertal	OW
LOH002C	1	1	F	0.70	Prepubertal	OW
LOH003C	1	1	M	NA	NA	NW (incl UW)
LOH003C	1	1	M	NA	NA	NW (incl UW)
LOH003C	1	1	M	NA	NA	NW (incl UW)
LOH004C	1	1	M	0.33	Prepubertal	NW (incl UW)

1-10 of 12 rows | 1-7 of 9 columns   Previous 1 2 Next

```
##First analysis will not include interactions with sociodemographic variables
l.bf=longbf%>%dplyr::select(BF_ROC, when, wave, schoolid, id)
l.bf=na.omit(longbf)
```

```
#using a model that does not account for heteroscedasticity
mod.bf1.1= lme(BF_ROC*365 ~ when + wave ,
           random = ~1|schoolid/id,
           control=lmeControl(maxIter = 5000),
           data=l.bf,
           contrasts = list(when=cMat))
#plot showing that there is a lot more variation in the residuals during holidays vs school (b
lack points show holidays, they are spread out a lot more vertically than school (green and re
d))
E<-resid(mod.bf1.1,type="normalized")
Fit=fitted(mod.bf1.1)
op<-par(mfrow=c(1,2))
plot(x=Fit,y=E,xlab="Fitted values",ylab="Residuals",
     main="%BF ROC: Residuals vs fitted", col=l.bf$when)
```

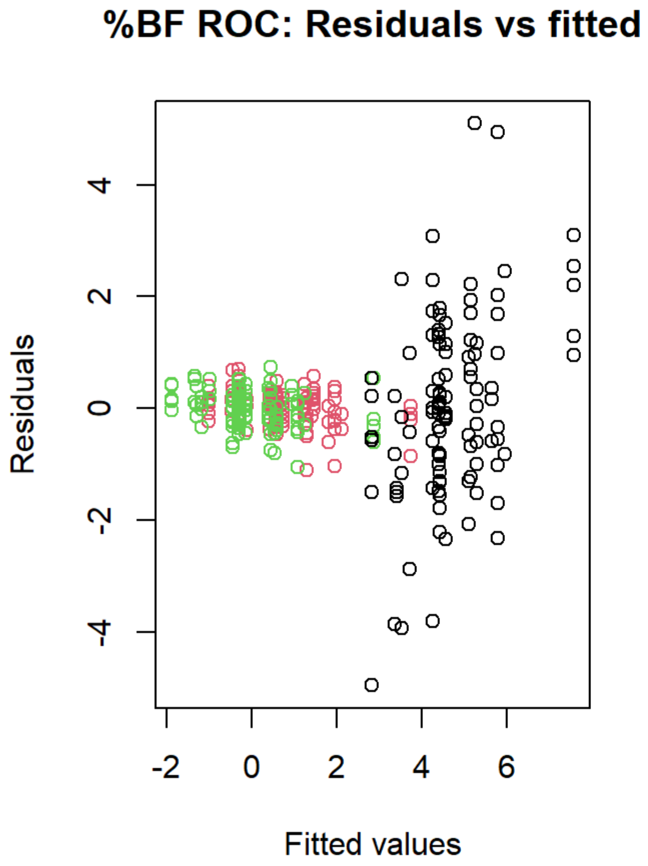


```
#now, accounting for different variance across time points
mod.bf1= lme(BF_ROC*365 ~ when + wave ,
           random = ~1|schoolid/id,
           weights = varIdent(form = ~1| when),method = "REML",
           control=lmeControl(maxIter = 5000),
           data=l.bf,
           contrasts = list(when=cMat))
#below plot shows how the adjustment has dealt with the heteroscedasticity
E<-resid(mod.bf1,type="normalized")
Fit=fitted(mod.bf1)
```

```
op<-par(mfrow=c(1,2))
plot(x=Fit,y=E,xlab="Fitted values",ylab="Residuals",
     main="%BF ROC: Residuals vs fitted", col=l.bf$when)
```

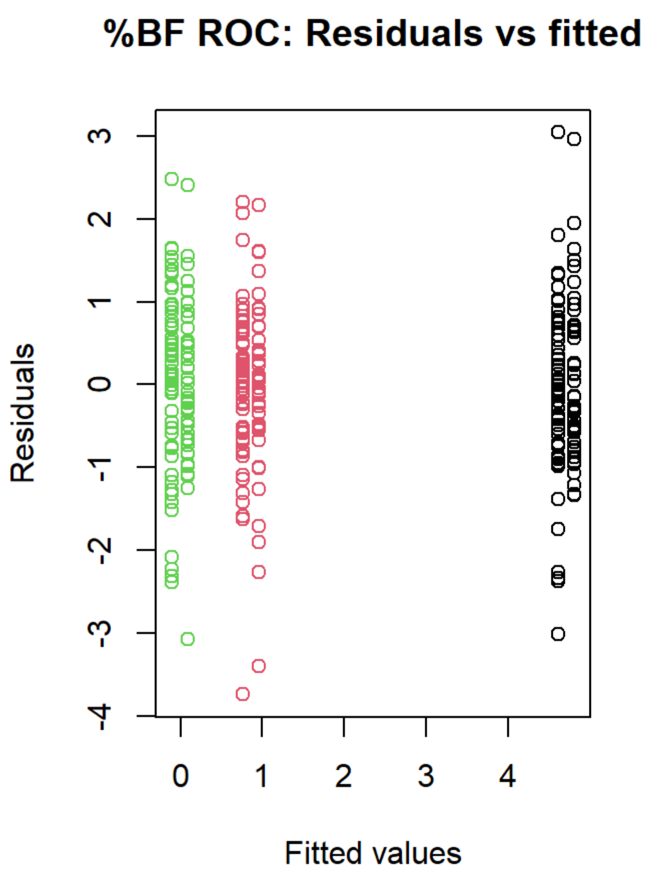


```
##########################################################################
#zBMI
zbmidata = d %>% dplyr::select(id, wave, schoolid, sex, sep, Puberty, bmicat.t1,
                      school1="dd zBMI T2-T1"  , school2="dd zBMI T5-T4" , holidays="dd zBMI H
ols")

#set up factors
zbmidata$wave=as.factor(zbmidata$wave)
zbmidata$schoolid=as.factor(zbmidata$schoolid)
zbmidata$sex=as.factor(zbmidata$sex)
zbmidata$Puberty=as.factor(zbmidata$Puberty)
zbmidata$id = as.factor(zbmidata$id)
zbmidata$bmicat.t1=as.factor(zbmidata$bmicat.t1)
zbmidata$sex=relevel(zbmidata$sex, ref="F")
zbmidata$Puberty=relevel(zbmidata$Puberty, ref="Midpubertal")
zbmidata$bmicat.t1=relevel(zbmidata$bmicat.t1, ref="NW (incl UW)")

longzbmi = gather(zbmidata, when, zBMI_ROC, school1:holidays)
longzbmi$when=as.factor(longzbmi$when)
longzbmi$when=relevel(longzbmi$when, ref="holidays")
l.zBMI=na.omit(longzbmi)
```

```
mod.zbmi1= lme(zBMI_ROC*365 ~ when + wave,
          random = ~1|schoolid/id,
          weights = varIdent(form = ~1| when),method = "REML",
          control=lmeControl(maxIter = 5000),
          data=l.zBMI,
          contrasts = list(when=cMat))

########################################################################
#WHT ratio
WHtdata = d %>% dplyr::select(id, wave, schoolid, sex, sep, Puberty, bmicat.t1,
                      school1="dd WHt T2-T1"  , school2="dd WHt T5-T4" , holidays="dd WHt Hols
")
WHtdata$wave=as.factor(WHtdata$wave)
WHtdata$schoolid=as.factor(WHtdata$schoolid)
WHtdata$sex=as.factor(WHtdata$sex)
WHtdata$Puberty=as.factor(WHtdata$Puberty)
WHtdata$id = as.factor(WHtdata$id)
WHtdata$bmicat.t1=as.factor(WHtdata$bmicat.t1)
WHtdata$sex=relevel(WHtdata$sex, ref="F")
WHtdata$Puberty=relevel(WHtdata$Puberty, ref="Midpubertal")
WHtdata$bmicat.t1=relevel(WHtdata$bmicat.t1, ref="NW (incl UW)")

longWHt = gather(WHtdata, when, WHt_ROC, school1:holidays)
longWHt$when=as.factor(longWHt$when)
longWHt$when=relevel(longWHt$when, ref="holidays")
l.WHt=na.omit(longWHt)

mod.WHt1= lme(WHt_ROC*100*365 ~ when+ wave,
          random = ~1|schoolid/id,
          weights = varIdent(form = ~1| when),method = "REML",
          control=lmeControl(maxIter = 5000),
          data=l.WHt,
          contrasts = list(when=cMat))

########################################################################
#SBJ
SBJdata = d %>% dplyr::select(id, wave, schoolid, sex, sep, Puberty, bmicat.t1,
                      school1="dd SBJ T2-T1"  , school2="dd SBJ T5-T4" , holidays="dd SBJ Hols
")
SBJdata$wave=as.factor(SBJdata$wave)
SBJdata$schoolid=as.factor(SBJdata$schoolid)
SBJdata$sex=as.factor(SBJdata$sex)
SBJdata$Puberty=as.factor(SBJdata$Puberty)
SBJdata$id = as.factor(SBJdata$id)
SBJdata$bmicat.t1=as.factor(SBJdata$bmicat.t1)
SBJdata$sex=relevel(SBJdata$sex, ref="F")
SBJdata$Puberty=relevel(SBJdata$Puberty, ref="Midpubertal")
SBJdata$bmicat.t1=relevel(SBJdata$bmicat.t1, ref="NW (incl UW)")

longSBJ = gather(SBJdata, when, SBJ_ROC, school1:holidays)
longSBJ$when=as.factor(longSBJ$when)
longSBJ$when=relevel(longSBJ$when, ref="holidays")
l.SBJ=na.omit(longSBJ)
```

```
mod.SBJ1= lme(SBJ_ROC*365 ~ when+ wave,
          random = ~1|schoolid/id,
          weights = varIdent(form = ~1| when),method = "REML",
          control=lmeControl(maxIter = 5000),
          data=l.SBJ,
          contrasts = list(when=cMat))

########################################################################
#VO2max
VO2maxdata = d %>% dplyr::select(id, wave, schoolid, sex, sep, Puberty, bmicat.t1,
                      school1=`dd VO2maxT2-T1`  , school2=`dd VO2max T5-T4` ,
                      holidays="dd VO2max Hols")
VO2maxdata$wave=as.factor(VO2maxdata$wave)
VO2maxdata$schoolid=as.factor(VO2maxdata$schoolid)
VO2maxdata$sex=as.factor(VO2maxdata$sex)
VO2maxdata$Puberty=as.factor(VO2maxdata$Puberty)
VO2maxdata$id = as.factor(VO2maxdata$id)
VO2maxdata$bmicat.t1=as.factor(VO2maxdata$bmicat.t1)
VO2maxdata$sex=relevel(VO2maxdata$sex, ref="F")
VO2maxdata$Puberty=relevel(VO2maxdata$Puberty, ref="Midpubertal")
VO2maxdata$bmicat.t1=relevel(VO2maxdata$bmicat.t1, ref="NW (incl UW)")

longVO2max = gather(VO2maxdata, when, VO2max_ROC, school1:holidays)
longVO2max$when=as.factor(longVO2max$when)
longVO2max$when=relevel(longVO2max$when, ref="holidays")
l.VO2max=na.omit(longVO2max)

mod.VO2max1= lme(VO2max_ROC*365 ~ when+ wave,
          random = ~1|schoolid/id,
          weights = varIdent(form = ~1| when),method = "REML",
          control=lmeControl(maxIter = 5000),
          data=l.VO2max,
          contrasts = list(when=cMat))

##model summaries
tab_model(mod.bf1, mod.zbmi1, mod.WHt1, mod.SBJ1, mod.VO2max1,
          digits=2, digits.p=3, collapse.ci = TRUE, show.r2 = FALSE,
          show.intercept=FALSE,
          rm.terms = "wave [2]")
```

	**BF_ROC * 365**		**zBMI_ROC * 365**		**WHt_ROC * 100 * 365**		**SBJ_ROC * 365**		**VO2max_ROC * 365**	
Predictors	*Estimates*	*p*	*Estimates*	*p*	*Estimates*	*p*	*Estimates*	*p*	*Estimates*	*p*
when [1]	3.85 (-0.40 – 8.09)	0.076	0.16 (-0.34 – 0.66)	0.535	-0.26 (-4.43 – 3.90)	0.901	17.19 (-4.27 – 38.65)	0.116	-4.71 (-8.26 – -1.15)	**0.010**
when [2]	4.71 (0.45 – 8.97)	**0.030**	0.16 (-0.34 – 0.66)	0.523	-0.36 (-4.55 – 3.83)	0.865	10.60 (-10.85 – 32.05)	0.331	-4.43 (-8.01 – -0.86)	**0.015**

Random Effects

σ^2	13.63	0.19	16.63	293.01	16.11
τ_{00}	0.00 $_{id}$	0.00 $_{id}$	0.85 $_{id}$	3.40 $_{id}$	1.15 $_{id}$
	0.00 $_{schoolid}$	0.00 $_{schoolid}$	0.00 $_{schoolid}$	0.00 $_{schoolid}$	0.00 $_{schoolid}$
N	107 $_{id}$	104 $_{id}$	187 $_{id}$	119 $_{id}$	101 $_{id}$
	19 $_{schoolid}$	19 $_{schoolid}$	22 $_{schoolid}$	19 $_{schoolid}$	19 $_{schoolid}$
Observations	321	312	397	357	303

These are the values for Table 3 in the paper

Results from Mixed Models accounting for repeated measures within ID, within schools, within study waves.

NB The assumption of homogeneity of variance has been relaxed using the varIdent function in nlme

When [1] is Holidays compared to Grade 4 When [2] is Holidays compared to Grade 5

```
#body fat
longbf1=na.omit(longbf)
mod.bf.int= lme(BF_ROC*365 ~ when*sex + when*sep + when*Puberty + when*bmicat.t1 + wave,
          random = ~1|schoolid/id,
          weights = varIdent(form = ~1| when),method = "REML",
          control=lmeControl(maxIter = 5000),
          data=longbf1,
          contrasts = list(when=cMat))

#zbmi
longzbmi1=na.omit(longzbmi)
mod.zbmi.int= lme(zBMI_ROC*365 ~ when*sex + when*sep + when*Puberty + when*bmicat.t1 + wave,
          random = ~1|schoolid/id,
          weights = varIdent(form = ~1| when),method = "REML",
          control=lmeControl(maxIter = 5000),
          data=longzbmi1,
          contrasts = list(when=cMat))



#WHT
longWHt1=na.omit(longWHt)
mod.WHt.int= lme(WHt_ROC*100*365 ~ when*sex + when*sep + when*Puberty + when*bmicat.t1 + wave,
          random = ~1|schoolid/id,
          weights = varIdent(form = ~1| when),method = "REML",
          control=lmeControl(maxIter = 5000),
          data=longWHt1,
          contrasts = list(when=cMat))



#SBJ
longSBJ1=na.omit(longSBJ)
mod.SBJ.int= lme(SBJ_ROC*365 ~ when*sex + when*sep + when*Puberty + when*bmicat.t1 + wave,
          random = ~1|schoolid/id,
          weights = varIdent(form = ~1| when),method = "REML",
          control=lmeControl(maxIter = 5000),
          data=longSBJ1,
          contrasts = list(when=cMat))
```

```
#VO2max
longVO2max1=na.omit(longVO2max)
mod.VO2max.int= lme(VO2max_ROC*365 ~ when*sex + when*sep + when*Puberty + when*bmicat.t1 + wav
e,
          random = ~1|schoolid/id,
          weights = varIdent(form = ~1| when),method = "REML",
          control=lmeControl(maxIter = 5000),
          data=longVO2max1,
          contrasts = list(when=cMat))
```

These are the values for Table 4 in the paper

Results from Mixed Models accounting for repeated measures within ID, within schools, within study waves. Interactions with covariates are included

NB The assumption of homogeneity of variance has been relaxed using the varIdent function in nlme

When [1] is Holidays compared to Grade 4 When [2] is Holidays compared to Grade 5

	**BF_ROC * 365**		**zBMI_ROC * 365**		**WHt_ROC * 100 * 365**		**SBJ_ROC * 365**		**VO2max_ROC * 365**	
Predictors	*Estimates*	*p*	*Estimates*	*p*	*Estimates*	*p*	*Estimates*	*p*	*Estimates*	*p*
when [1] × sex [M]	-1.97 (-14.10 – 10.16)	0.749	0.62 (-0.94 – 2.18)	0.433	-8.19 (-20.63 – 4.26)	0.196	-14.19 (-84.70 – 56.32)	0.692	7.36 (-3.44 – 18.16)	0.181
when [2] × sex [M]	-3.73 (-15.91 – 8.44)	0.546	0.31 (-1.24 – 1.87)	0.691	-7.01 (-19.55 – 5.53)	0.272	-14.58 (-85.08 – 55.92)	0.684	8.05 (-2.81 – 18.91)	0.145
when [1] × sep	-7.49 (-14.82 – -0.16)	**0.045**	-0.68 (-1.56 – 0.20)	0.127	-1.46 (-8.69 – 5.77)	0.692	-4.99 (-41.41 – 31.44)	0.788	-0.62 (-6.55 – 5.31)	0.837
when [2] × sep	-9.44 (-16.79 – -2.08)	**0.012**	-0.92 (-1.80 – -0.04)	**0.040**	-2.89 (-10.19 – 4.41)	0.436	-6.12 (-42.54 – 30.29)	0.741	-2.64 (-8.60 – 3.32)	0.383
when [1] × Puberty [Early Pubertal]	-12.83 (-23.91 – -1.75)	**0.023**	-1.36 (-2.70 – -0.02)	**0.046**	-12.01 (-23.24 – -0.79)	**0.036**	-9.23 (-65.04 – 46.58)	0.745	-5.36 (-14.62 – 3.89)	0.254
when [2] × Puberty [Early Pubertal]	-10.81 (-21.93 – 0.31)	0.057	-1.21 (-2.55 – 0.13)	0.077	-10.84 (-22.18 – 0.49)	0.061	3.30 (-52.51 – 59.11)	0.907	-4.63 (-13.93 – 4.68)	0.328
when [1] × Puberty [Prepubertal]	0.17 (-12.99 – 13.34)	0.979	-0.47 (-2.13 – 1.20)	0.582	12.16 (-1.54 – 25.86)	0.082	49.96 (-25.58 – 125.51)	0.194	-12.90 (-24.50 – -1.30)	**0.030**
when [2] ×	3.15	0.639	-0.02	0.981	11.59	0.099	61.54	0.110	-13.18	**0.027**

Puberty [Prepubertal]	(-10.07 – 16.36)		(-1.68 – 1.64)		(-2.22 – 25.40)		(-13.99 – 137.07)		(-24.84 – -1.51)	
when [1] × bmicat t1 [OW]	11.23 (1.31 – 21.15)	**0.027**	0.90 (-0.29 – 2.09)	0.139	3.10 (-7.06 – 13.27)	0.548	3.48 (-50.24 – 57.20)	0.899	-11.30 (-20.24 – -2.37)	**0.013**
when [2] × bmicat t1 [OW]	11.87 (1.91 – 21.82)	**0.020**	0.93 (-0.26 – 2.12)	0.127	3.11 (-7.13 – 13.35)	0.550	5.86 (-47.85 – 59.57)	0.830	-11.78 (-20.76 – -2.79)	**0.010**
when [1] × bmicat t1 [OB]	4.36 (-9.21 – 17.93)	0.527	0.19 (-1.44 – 1.81)	0.822	4.93 (-8.59 – 18.46)	0.473	8.77 (-62.24 – 79.79)	0.808	-3.95 (-17.13 – 9.22)	0.555
when [2] × bmicat t1 [OB]	6.12 (-7.50 – 19.74)	0.377	0.42 (-1.21 – 2.04)	0.613	7.09 (-6.54 – 20.73)	0.306	7.95 (-63.06 – 78.96)	0.826	-7.81 (-21.06 – 5.43)	0.246
**Random Effects**										
σ^2	12.71		0.18		16.36		289.64		16.26	
τ_{00}	0.00 id		0.04 id		0.83 id		3.33 id		1.21 id	
	0.00 schoolid		0.00 schoolid		0.00 schoolid		0.00 schoolid		0.00 schoolid	
N	107 id		104 id		187 id		119 id		101 id	
	19 schoolid		19 schoolid		22 schoolid		19 schoolid		19 schoolid	
Observations	321		312		397		357		303	

Here are some plots that show conditional Means for Annualised Rates of Change, as predicted from multi-level models with interaction effects between all covariates and time point.

```
#BF
coefs.bf=coef(summary(mod.bf.int))
coefs.bf=data.frame(coefs.bf[11:22,1:2])
coefs.bf = coefs.bf %>% mutate(lowCI = Value- 1.96*Std.Error)
coefs.bf = coefs.bf %>% mutate(upCI = Value+ 1.96*Std.Error)
coefs.bf$factor = c("Sex","Sex", "SEP zscore","SEP zscore",
                    "Puberty","Puberty","Puberty","Puberty",
                    "BMI cat","BMI cat","BMI cat","BMI cat")
coefs.bf$level = c("Male","Male","+1SD SEP", "+1SD SEP",
                   "Early Puberty", "Early Puberty",
                   "Prepubertal","Prepubertal",
                   "Overweight","Overweight","Obesity","Obesity")
coefs.bf$Comparison_vs_Holidays = rep(c("Grade 4", "Grade 5"),6)
coefs.bf$Effect = coefs.bf$Value
coefs.bf$level=factor(coefs.bf$level,
                      levels=c("Male","+1SD SEP",  "Prepubertal",
                    "Early Puberty","Overweight","Obesity"))
(bf.plot=ggplot(coefs.bf, aes(x=level, y=Effect))+
  geom_point(aes(colour=Comparison_vs_Holidays),size=2, position = position_dodge(width = 0.2)
)+
  geom_errorbar(aes(ymin=lowCI, ymax=upCI, colour=Comparison_vs_Holidays),
```

```
                width=.2, size=0.8, position = position_dodge(width = 0.2))+
  geom_hline(yintercept = 0, linetype="dashed", color = "black")+
  annotate("segment", x=0.6, y=-22, xend=0.6, yend=22,
               col="grey26", arrow=arrow(length=unit(0.3, "cm"), ends="both")) +
  annotate("text", x=0.2, y=-11, label="Lower RoC during holidays \n in displayed group (bold)
 \n vs comparator group", angle=90, colour="grey26",  size=2.5)+
  annotate("text", x=0.2, y=11, label="Higher RoC during holidays \n in displayed group (bold)
 \n vs comparator group", angle=90, colour="grey26", size=2.5)+
  coord_cartesian(xlim = c(0.5, 6), ylim=c(-22.5,21), clip="off") +
  theme(axis.text = element_text(face="bold", size=7),
        plot.title = element_text(hjust = 0.5),
        panel.background = element_rect(fill='white', colour='black'),
        panel.grid.major.y = element_line(colour = "grey88"))+
annotate(geom = "text", x = c(1,2,3.5,5.5), y = rep(-27.5,4),
           label = c("vs Female","vs mean SEP",
                     "vs Midpubertal",
                     "vs Normal weight"),
           vjust = 0, size=2.3, colour="grey26")+
    annotate(geom = "text", x = c(1.95, 2.05, 3.95, 4.95, 5.05), y = c(0.4,-1.5, -1.3, 21.7,22
.2 ),
           label = rep("*",5), size=5, colour="black")+
  ggtitle("Anualised Change in Body Fat%")+
  xlab("")+
  ylab("Model estimate for effect size"))
```

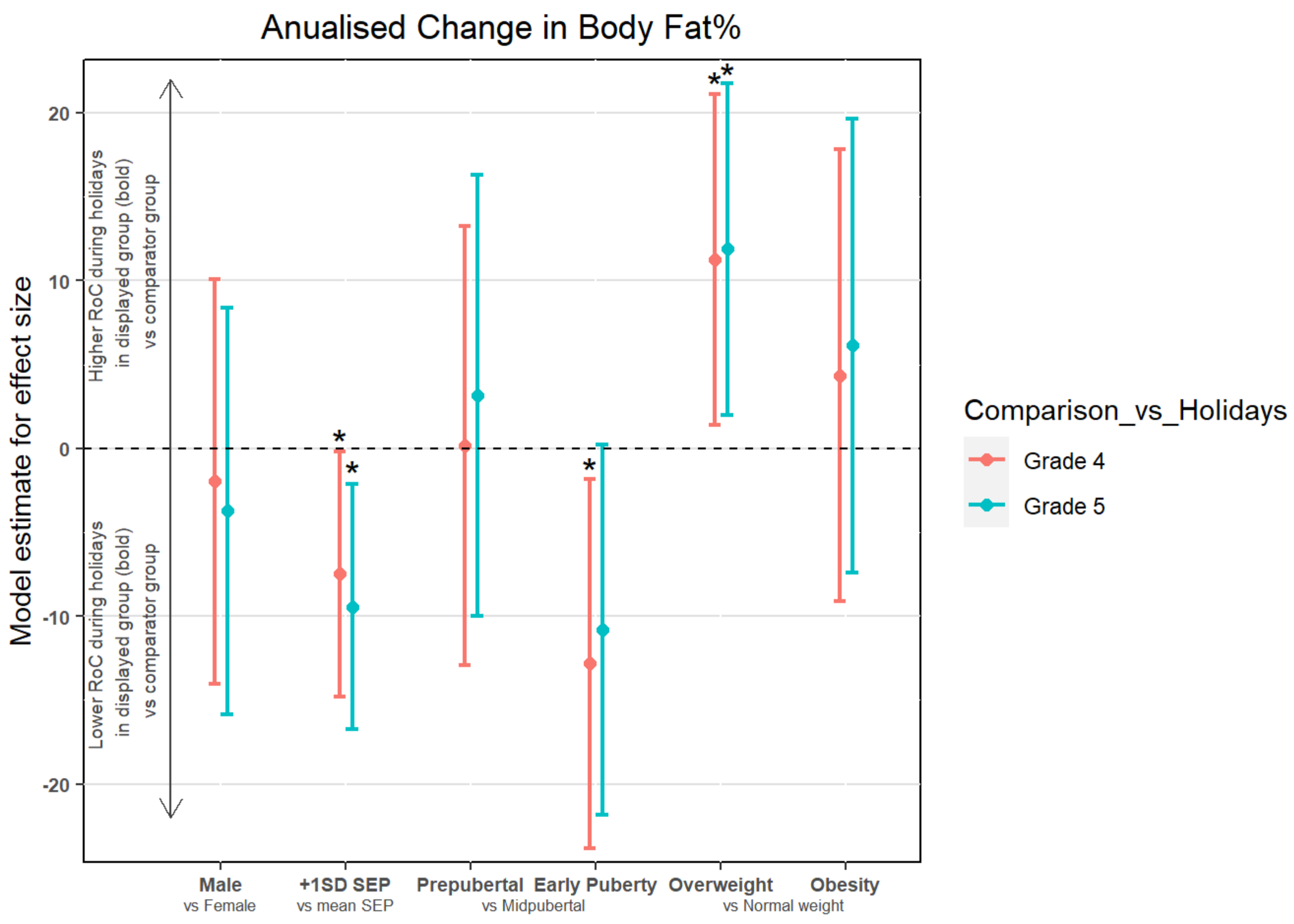


```
#zbmi
```

```
coefs.zbmi=coef(summary(mod.zbmi.int))
coefs.zbmi=data.frame(coefs.zbmi[11:22,1:2])
coefs.zbmi = coefs.zbmi %>% mutate(lowCI = Value- 1.96*Std.Error)
coefs.zbmi = coefs.zbmi %>% mutate(upCI = Value+ 1.96*Std.Error)
coefs.zbmi$factor = c("Sex","Sex", "SEP zscore","SEP zscore",
                    "Puberty","Puberty","Puberty","Puberty",
                    "BMI cat","BMI cat","BMI cat","BMI cat")
coefs.zbmi$level = c("Male","Male","+1SD SEP", "+1SD SEP",
                       "Early Puberty", "Early Puberty",
                       "Prepubertal","Prepubertal",
                       "Overweight","Overweight","Obesity","Obesity")
coefs.zbmi$Comparison_vs_Holidays = rep(c("Grade 4", "Grade 5"),6)
coefs.zbmi$Effect = coefs.zbmi$Value
coefs.zbmi$level=factor(coefs.zbmi$level,
                          levels=c("Male","+1SD SEP",  "Prepubertal",
                    "Early Puberty","Overweight","Obesity"))
(zbmi.plot=ggplot(coefs.zbmi, aes(x=level, y=Effect))+
  geom_point(aes(colour=Comparison_vs_Holidays),size=2, position = position_dodge(width = 0.2)
)+
  geom_errorbar(aes(ymin=lowCI, ymax=upCI, colour=Comparison_vs_Holidays),
                width=.2, size=0.8, position = position_dodge(width = 0.2))+
  geom_hline(yintercept = 0, linetype="dashed", color = "black")+
  annotate("segment", x=0.6, y=-3, xend=0.6, yend=3,
               col="grey26", arrow=arrow(length=unit(0.3, "cm"), ends="both")) +
  annotate("text", x=0.2, y=-1.5, label="Lower RoC during holidays \n in displayed group (bold
) \n vs comparator group", angle=90, colour="grey26", size=2.5)+
  annotate("text", x=0.2, y=1.5, label="Higher RoC during holidays \n in displayed group (bold
) \n vs comparator group", angle=90, colour="grey26", size=2.5)+
  coord_cartesian(xlim = c(0.5, 6), ylim=c(-3,3), clip="off") +
  theme(axis.text = element_text(face="bold", size=7),
        plot.title = element_text(hjust = 0.5),
        panel.background = element_rect(fill='white', colour='black'),
        panel.grid.major.y = element_line(colour = "grey88"))+
          scale_y_continuous(breaks=seq(-2,2,1))+
 annotate(geom = "text", x = c(1,2,3.5,5.5), y = rep(-3.7,4),
          label = c("vs Female","vs mean SEP",
                       "vs Midpubertal",
                       "vs Normal weight"),
          vjust = 0, size=2.3, colour="grey26")+
    annotate(geom = "text", x = c(2.05, 3.95), y = c(0.05, 0.05),
          label = rep("*",2), size=5, colour="black")+
  ggtitle("Anualised Change in zBMI")+
  xlab("")+
  ylab("Model estimate for effect size"))
```

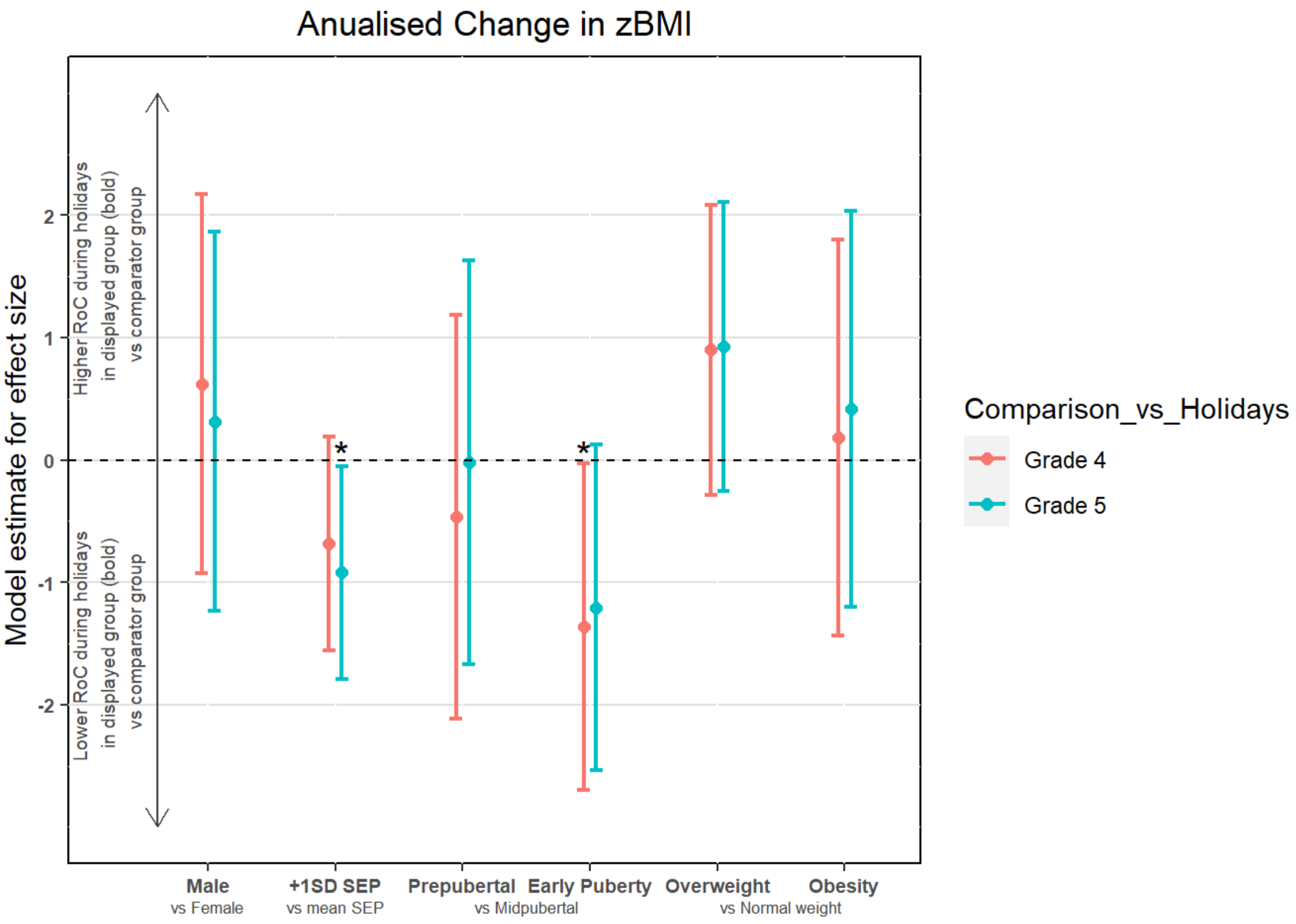


```
#WHt
coefs.WHt=coef(summary(mod.WHt.int))
coefs.WHt=data.frame(coefs.WHt[11:22,1:2])
coefs.WHt = coefs.WHt %>% mutate(lowCI = Value- 1.96*Std.Error)
coefs.WHt = coefs.WHt %>% mutate(upCI = Value+ 1.96*Std.Error)
coefs.WHt$factor = c("Sex","Sex", "SEP zscore","SEP zscore",
                    "Puberty","Puberty","Puberty","Puberty",
                    "BMI cat","BMI cat","BMI cat","BMI cat")
coefs.WHt$level = c("Male","Male","+1SD SEP", "+1SD SEP",
                       "Early Puberty", "Early Puberty",
                       "Prepubertal","Prepubertal",
                       "Overweight","Overweight","Obesity","Obesity")
coefs.WHt$Comparison_vs_Holidays = rep(c("Grade 4", "Grade 5"),6)
coefs.WHt$Effect = coefs.WHt$Value
coefs.WHt$level=factor(coefs.WHt$level,
                          levels=c("Male","+1SD SEP",  "Prepubertal",
                    "Early Puberty","Overweight","Obesity"))
(WHt.plot=ggplot(coefs.WHt, aes(x=level, y=Effect))+
  geom_point(aes(colour=Comparison_vs_Holidays),size=2, position = position_dodge(width = 0.2)
)+
  geom_errorbar(aes(ymin=lowCI, ymax=upCI, colour=Comparison_vs_Holidays),
                width=.2, size=0.8, position = position_dodge(width = 0.2))+
  geom_hline(yintercept = 0, linetype="dashed", color = "black")+
  annotate("segment", x=0.6, y=-22, xend=0.6, yend=22,
               col="grey26", arrow=arrow(length=unit(0.3, "cm"), ends="both")) +
  annotate("text", x=0.2, y=-11, label="Lower RoC during holidays \n in displayed group (bold)
```

```
\n vs comparator group", angle=90, colour="grey26", size=2.5)+
  annotate("text", x=0.2, y=11, label="Higher RoC during holidays \n in displayed group (bold)
\n vs comparator group", angle=90, colour="grey26", size=2.5)+
  coord_cartesian(xlim = c(0.5, 6), ylim=c(-22.5,25), clip="off") +
  theme(axis.text = element_text(face="bold", size=7),
        plot.title = element_text(hjust = 0.5),
        panel.background = element_rect(fill='white', colour='black'),
        panel.grid.major.y = element_line(colour = "grey88"))+
  annotate(geom = "text", x = c(1,2,3.5,5.5), y = rep(-28,4),
           label = c("vs Female","vs mean SEP",
                     "vs Midpubertal",
                     "vs Normal weight"),
           vjust = 0, size=2.3, colour="grey26")+
    annotate(geom = "text", x = c(3.95), y = c(0.5),
           label = rep("*",1), size=5, colour="black")+
  ggtitle("Anualised Change in WHt%")+
  xlab("")+
  ylab("Model estimate for effect size"))
```

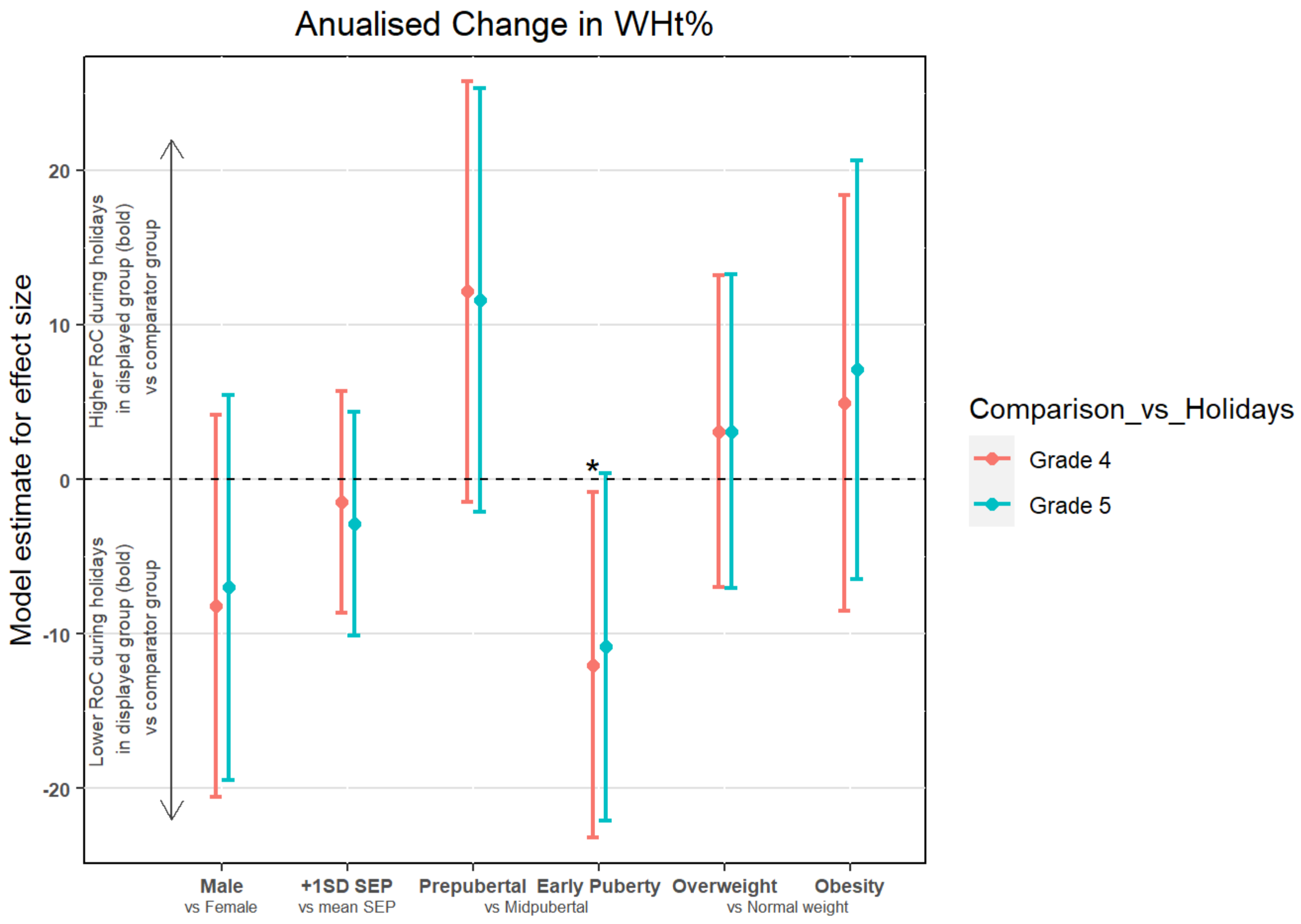


```
#SBJ
coefs.SBJ=coef(summary(mod.SBJ.int))
coefs.SBJ=data.frame(coefs.SBJ[11:22,1:2])
coefs.SBJ = coefs.SBJ %>% mutate(lowCI = Value- 1.96*Std.Error)
coefs.SBJ = coefs.SBJ %>% mutate(upCI = Value+ 1.96*Std.Error)
coefs.SBJ$factor = c("Sex","Sex", "SEP zscore","SEP zscore",
                    "Puberty","Puberty","Puberty","Puberty",
```

```
                    "BMI cat","BMI cat","BMI cat","BMI cat")
coefs.SBJ$level = c("Male","Male","+1SD SEP", "+1SD SEP",
                       "Early Puberty", "Early Puberty",
                       "Prepubertal","Prepubertal",
                       "Overweight","Overweight","Obesity","Obesity")
coefs.SBJ$Comparison_vs_Holidays = rep(c("Grade 4", "Grade 5"),6)
coefs.SBJ$Effect = coefs.SBJ$Value
coefs.SBJ$level=factor(coefs.SBJ$level,
                          levels=c("Male","+1SD SEP",  "Prepubertal",
                    "Early Puberty","Overweight","Obesity"))
(SBJ.plot=ggplot(coefs.SBJ, aes(x=level, y=Effect))+
  geom_point(aes(colour=Comparison_vs_Holidays),size=2, position = position_dodge(width = 0.2)
)+
  geom_errorbar(aes(ymin=lowCI, ymax=upCI, colour=Comparison_vs_Holidays),
                width=.2, size=0.8, position = position_dodge(width = 0.2))+
  geom_hline(yintercept = 0, linetype="dashed", color = "black")+
  annotate("segment", x=0.6, y=-70, xend=0.6, yend=80,
               col="grey26", arrow=arrow(length=unit(0.3, "cm"), ends="both")) +
  annotate("text", x=0.2, y=-50, label="Lower RoC during holidays \n in displayed group (bold)
 \n vs comparator group", angle=90, colour="grey26", size=2.5)+
  annotate("text", x=0.2, y=55, label="Higher RoC during holidays \n in displayed group (bold)
 \n vs comparator group", angle=90, colour="grey26", size=2.5)+
  coord_cartesian(xlim = c(0.5, 6), ylim=c(-82.5,130), clip="off") +
  theme(axis.text = element_text(face="bold", size=7),
        plot.title = element_text(hjust = 0.5),
        panel.background = element_rect(fill='white', colour='black'),
        panel.grid.major.y = element_line(colour = "grey88"))+
  annotate(geom = "text", x = c(1,2,3.5,5.5), y = rep(-107,4),
           label = c("vs Female","vs mean SEP",
                       "vs Midpubertal",
                       "vs Normal weight"),
           vjust = 0, size=2.3, colour="grey26")+
  ggtitle("Anualised Change in SBJ (cm)")+
  xlab("")+
  ylab("Model estimate for effect size"))
```

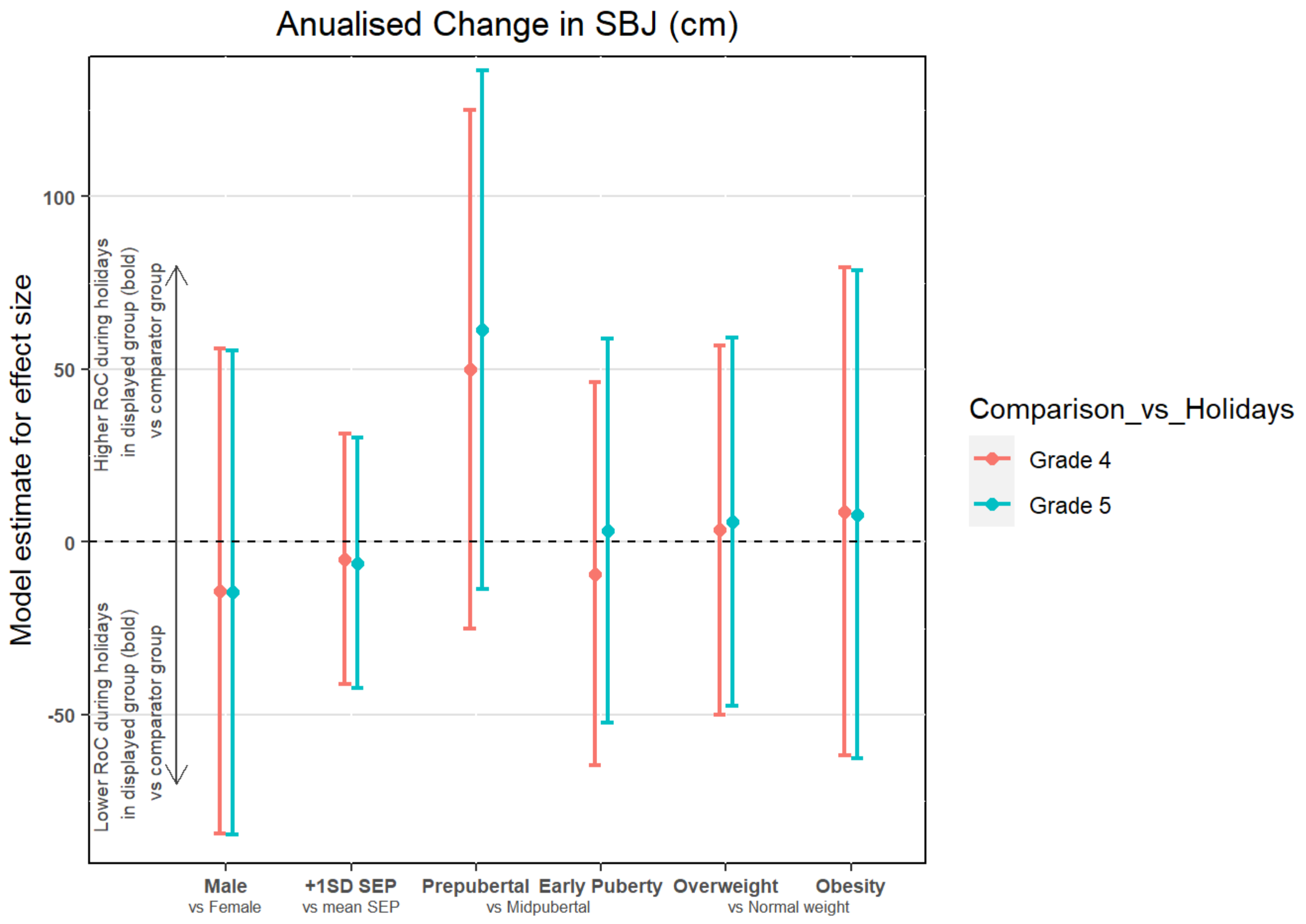


```
#VO2max
coefs.VO2max=coef(summary(mod.VO2max.int))
coefs.VO2max=data.frame(coefs.VO2max[11:22,1:2])
coefs.VO2max = coefs.VO2max %>% mutate(lowCI = Value- 1.96*Std.Error)
coefs.VO2max = coefs.VO2max %>% mutate(upCI = Value+ 1.96*Std.Error)
coefs.VO2max$factor = c("Sex","Sex", "SEP zscore","SEP zscore",
                    "Puberty","Puberty","Puberty","Puberty",
                    "BMI cat","BMI cat","BMI cat","BMI cat")
coefs.VO2max$level = c("Male","Male","+1SD SEP", "+1SD SEP",
                       "Early Puberty", "Early Puberty",
                       "Prepubertal","Prepubertal",
                       "Overweight","Overweight","Obesity","Obesity")
coefs.VO2max$Comparison_vs_Holidays = rep(c("Grade 4", "Grade 5"),6)
coefs.VO2max$Effect = coefs.VO2max$Value
coefs.VO2max$level=factor(coefs.VO2max$level,
                          levels=c("Male","+1SD SEP",  "Prepubertal",
                    "Early Puberty","Overweight","Obesity"))
(VO2max.plot=ggplot(coefs.VO2max, aes(x=level, y=Effect))+
  geom_point(aes(colour=Comparison_vs_Holidays),size=2, position = position_dodge(width = 0.2)
)+
  geom_errorbar(aes(ymin=lowCI, ymax=upCI, colour=Comparison_vs_Holidays),
                width=.2, size=0.8, position = position_dodge(width = 0.2))+
  geom_hline(yintercept = 0, linetype="dashed", color = "black")+
  annotate("segment", x=0.6, y=-18, xend=0.6, yend=18,
               col="grey26", arrow=arrow(length=unit(0.3, "cm"), ends="both")) +
  annotate("text", x=0.2, y=-11, label="Lower RoC during holidays \n in displayed group (bold)
```

```
\n vs comparator group", angle=90, colour="grey26", size=2.5)+
 annotate("text", x=0.2, y=11, label="Higher RoC during holidays \n in displayed group (bold)
\n vs comparator group", angle=90, colour="grey26", size=2.5)+
 coord_cartesian(xlim = c(0.5, 6), ylim=c(-24,21), clip="off") +
 theme(axis.text = element_text(face="bold", size=7),
       plot.title = element_text(hjust = 0.5),
       panel.background = element_rect(fill='white', colour='black'),
       panel.grid.major.y = element_line(colour = "grey88"))+
 annotate(geom = "text", x = c(1,2,3.5,5.5), y = rep(-29.5,4),
          label = c("vs Female","vs mean SEP",
                    "vs Midpubertal",
                    "vs Normal weight"),
          vjust = 0, size=2.3, colour="grey26")+
   annotate(geom = "text", x = c(2.95, 3.05, 4.95, 5.05), y = c(-0.8,-1.1, -2, -2.3),
          label = rep("*",4), size=5, colour="black")+
 ggtitle("Anualised Change in VO2max")+
 xlab("")+
 ylab("Model estimate for effect size"))
```

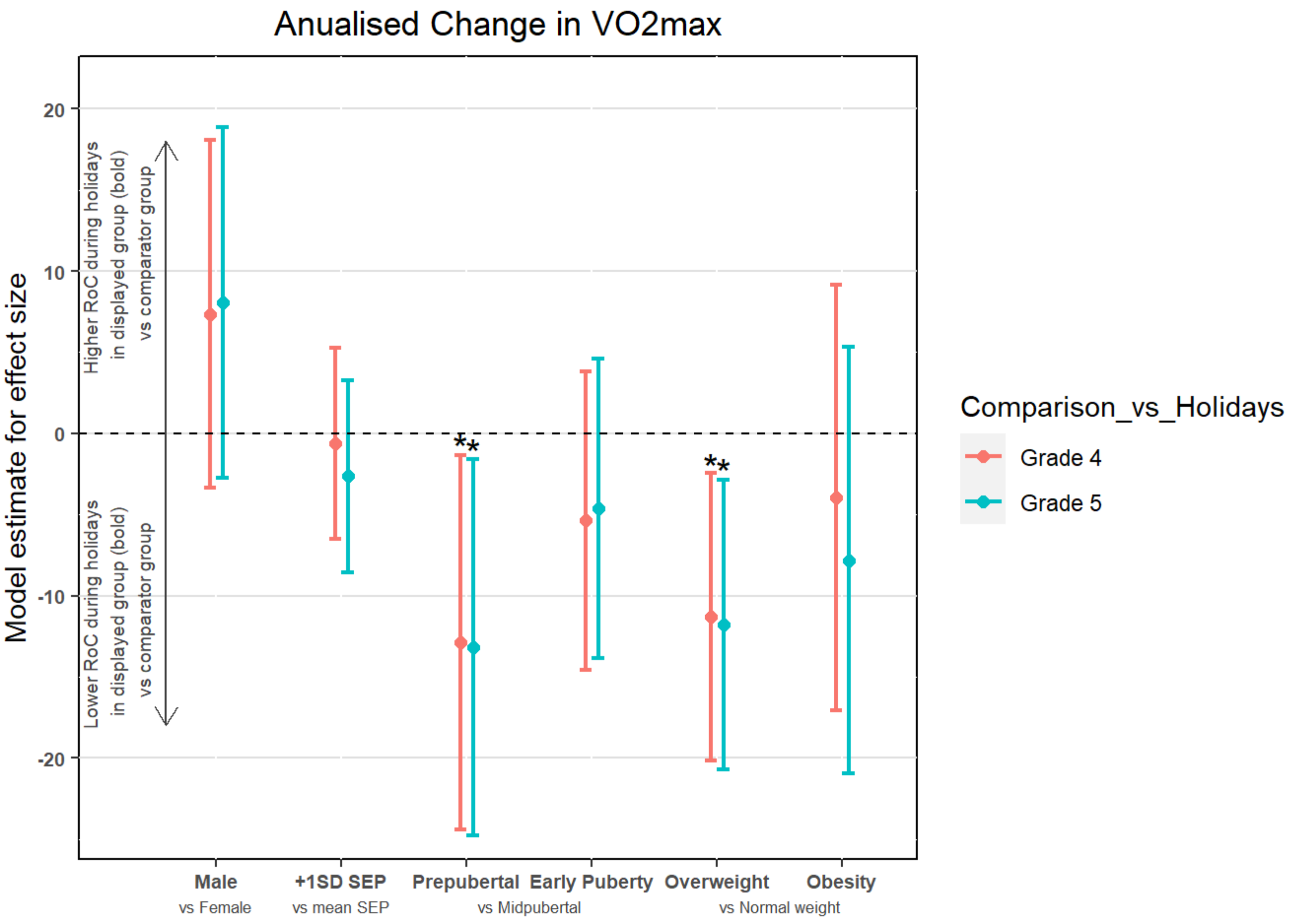

Supplement: Supplementary file 1 — Supplementary Material 1 [file 12889_2023_17009_MOESM1_ESM.pdf]
